# Mushroom Polysaccharide-Assisted Anticarcinogenic Mycotherapy: Reviewing Its Clinical Trials

**DOI:** 10.3390/molecules27134090

**Published:** 2022-06-25

**Authors:** Iyyakkannu Sivanesan, Manikandan Muthu, Judy Gopal, Jae-Wook Oh

**Affiliations:** 1 Department of Bioresources and Food Science, Institute of Natural Science and Agriculture, Konkuk University, 1 Hwayang-dong, Gwangjin-gu, Seoul 05029, Korea; siva74@konkuk.ac.kr; 2 Department of Research and Innovation, Saveetha School of Engineering, Saveetha Institute of Medical and Technical Sciences (SIMATS), Thandalam, Chennai 602105, Tamil Nadu, India; bhagatmani@gmail.com (M.M.); jejudy777@gmail.com (J.G.); 3 Department of Stem Cell and Regenerative Biotechnology, Konkuk University, 1 Hwayang-dong, Gwangjin-gu, Seoul 05029, Korea

**Keywords:** mushroom polysaccharides, anticancer, treatment, clinical trials, glucans, lentinans

## Abstract

Of the biologically active components, polysaccharides play a crucial role of high medical and pharmaceutical significance. Mushrooms have existed for a long time, dating back to the time of the Ancient Egypt and continue to be well explored globally and experimented with in research as well as in national and international cuisines. Mushroom polysaccharides have slowly become valuable sources of nutraceuticals which have been able to treat various diseases and disorders in humans. The application of mushroom polysaccharides for anticancer mycotherapy is what is being reviewed herein. The widespread health benefits of mushroom polysaccharides have been highlighted and the significant inputs of mushroom-based polysaccharides in anticancer clinical trials have been presented. The challenges and limitation of mushroom polysaccharides into this application and the gaps in the current application areas that could be the future direction have been discussed.

## 1. Introduction

Polysaccharides include structurally diverse macromolecules that occur most prevalently in nature. They are made up of repetitive structural features linked by glycosidic linkages. Polysaccharides can hold ample biological information owing to their high potential for structural variability [1]. In recent decades, the bioactivities of polysaccharides have gained a lot of attention. Polysaccharides are the most recognized and powerful mushroom-derived substance with a range of biologically important characteristics. Mushroom polysaccharides contribute much to the food and medicine industries and towards health applications, predominantly in China and Japan [1]. β-glucan is the most versatile mushroom polysaccharide because of its high biological potential. β-glucans consists of a glucose residue backbone associated with β-(1–3) glycosidic bonds, often connected by β-(1–6) linkages with side-chain glucose residues. Few are linked by β-(1–3), (1–6) glycosidic bonds and α-(1–3) glycosidic bonds, while most others are true heteroglycans [2,3,4,5,6,7].

**Figure 1:**
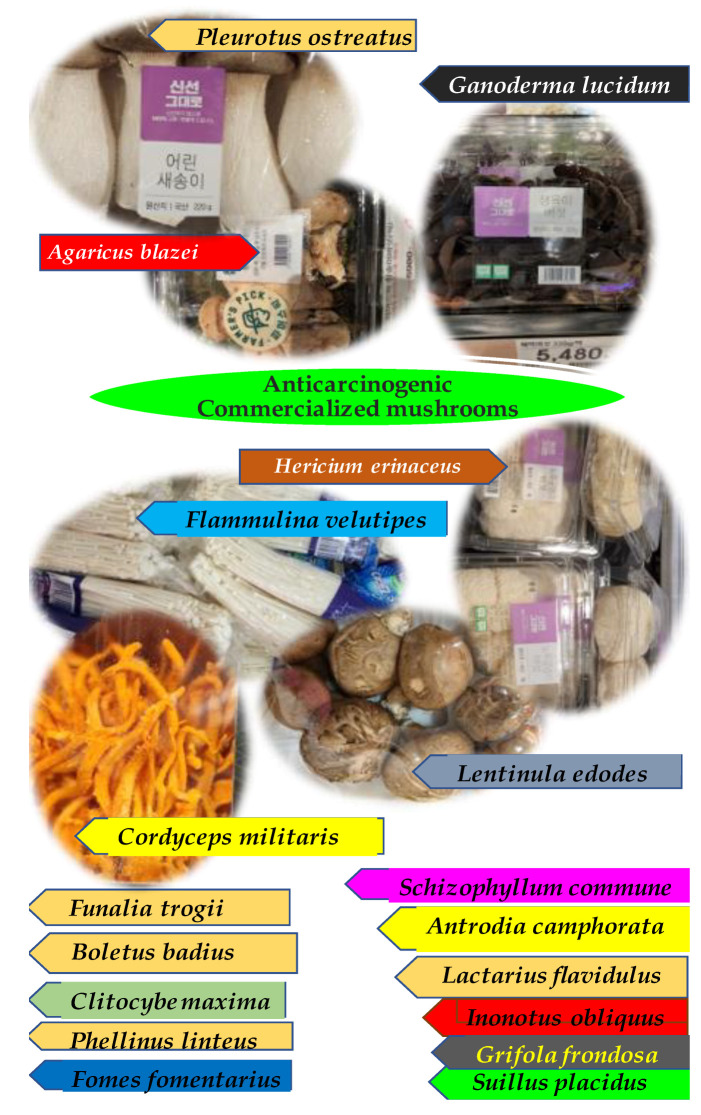
Snapshot of commercially available mushrooms in a traditional Asian market.

The most promising biopharmacological activities of mushroom polysaccharides are their immunomodulation and anticancer effects. The bioactive substances found in mushrooms are secondary metabolites such as acids, terpenoids, polyphenols, sesquiterpenes, alkaloids, lactones, sterols, metal chelating agents, nucleotide analogs and vitamins, glycoproteins and polysaccharides. In China, edible wild mushrooms, are appreciated as food and play pivotal roles in traditional Chinese medicine. The high protein, carbohydrate, essential mineral and low energy levels give mushrooms a nutritional value comparable with meat, eggs and milk. Figure 1 gives a snapshot of all edible mushrooms commercially predominating Asian freshmarkets.

Polysaccharides are structurally diverse class of versatile macromolecules [8]. Mushroom polysaccharides have been successfully applied in anticancer experiments. The extraction of mushroom polysaccharides starts with treating dried powdered mushroom with 80% ethanol to remove the low molecular weight substances. Crude fractions are further extracted with water, 1% ammonium oxalate and 5% sodium hydroxide. The extracted polysaccharides can be fractionally purified by the ethanol concentration, fractional precipitation, ion-exchange chromatography, gel filtration and affinity chromatography—all of which can be used individually or in combination. Yap and Ng [9] isolated β-glucan through ethanol precipitation, followed by freeze-drying in liquid nitrogen.

As it stands, mushrooms are documented as the main underutilized resource of nutritious foods, even though there is huge interest in the related area of cost-effective biotechnology. However there are approximately 1600 f mushroom species, and only 100 have been declared edible. Of the 33 edible species of mushrooms, only three species are commonly propagated worldwide, i.e., paddy straw mushroom (*Volvariella volvacea* L.), white button mushrooms (*Agaricus bisporus* L.) and oyster mushroom (*Pleurotus ostreatus* L.) [10].

When it comes to the bioactivity of mushrooms, there is voluminous documentation, with which we present the most significant anticarcinogenic mushrooms, as presented in Table 1. Such reports highlight the importance of the use of several mushrooms in anticancer applications.

The objective of the current review was to exclusively touch on the medicinal properties of mushroom polysaccharides and to review the advances made using mushroom polysaccharides in cancer therapy. This review elaborately discusses the clinical trials that have been documented using mushroom polysaccharides and the progress made through them. The challenges facing the further extrapolation of mushroom polysaccharides into cancer mycotherapy and the prospects into the future of mushroom polysaccharides are also discussed.

## 2. Medicinal Attributes of Mushroom Polysaccharides

The incorporation of mushrooms into our diet by Greeks and Romans has a long history dating back to the days of yore. Mushrooms were designated as the food of Gods by Romans and as an elixir of life by the Chinese. Many cultures have utilized them for centuries and now they are known for their ability to safeguard general health, as well as prevent and treat diseases owing to their immunomodulatory and antineoplastic properties. In the last decade, interest in the pharmaceutical potential of mushrooms has increased and there is widespread awareness among the educated public. Now, mushrooms are considered as mini-pharmaceutical factories that yield products with miraculous biological properties [34].

More than 100 medicinal attributes have been correlated with mushrooms, that can be broadly listed as having antioxidant, anticancer, antidiabetic, antiallergic, immunomodulating, cardiovascular protector, anticholesterolemic, antiviral, antibacterial, antiparasitic, antifungal, detoxification and hepatoprotective effects; additionally, they also protect against tumor development and inflammatory processes [3,4,5,6]. Numerous molecules synthesized by mushrooms are known to be bioactive. These bioactive compounds include polysaccharides, proteins, fats, tocopherols, phenolics, flavonoids, carotenoids, minerals, glycosides, alkaloids, volatile oils, terpenoids, folates, lectins, enzymes, ascorbic and organic acids.

**Figure 2:**
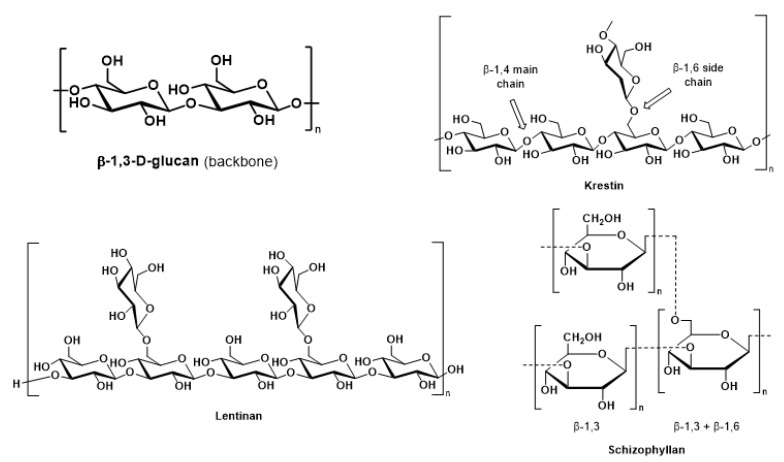
Chemical structures of anticarcinogenic mushroom polysaccharides.

In the context of oxidative stress, mushrooms have a reputation for their use in oriental medicine against numerous diseases. Currently, mushroom extracts are commercialized as dietary supplements for enhancing their immune function and antitumor activity [35,36,37,38,39,40,41,42,43]. Commercialized mushroom extract powers and capsules are on the market. A vast number of reviews have dealt with the medicinal aspects of mushrooms and their various bioactive components [44]. Figure 2 gives the molecular structures of the predominant mushroom polysaccharides applied in anticarcinogenic mycotherapy.

## 3. A Snapshot of the Application of Mushroom Polysaccharide-Based Cancer Mycotherapy

Polysaccharides that exhibit antitumor activity show significant variation in their chemical structure. Anticancer activity has been demonstrated by glycan types that include homopolymers as well as extremely complicated heteropolymers [45]. Anticarcinogenic and immunostimulating mushroom-based polysaccharides from the genera Basidiomycetes have been explored, and the primary structure of mushroom polysaccharides relies on features such as the sequence and their monosaccharide composition, position, configuration as well as the number of non-carbohydrate moieties.

Developing specific remedies against cancer is a challenging task resembling that of developing vaccines against viral infections and antibiotics. Cancerous cells originate from normal cells, and thus the world is seeking a drug that will specifically target and destroy the cancer cells without affecting the normal cells, which would be the ideal result during cancer therapy. This is a huge challenge for cancer researchers. Immunotherapy has been involved in targeting and removing cancer cells, immunopotentiators, immunoinitiators and biological response modulators (BRMs) that act against carcinogenesis and induce carcinostasis are sought after [46] and immunoceuticals [47]. Immunoceuticals manifest immunotherapeutic efficacy when administered orally, and mushroom polysaccharides fall under the category of immunoceuticals. More than 50 mushrooms species have yielded potential immunoceuticals that exhibit anticancer activity in vitro and in animal models. Mushrooms belong to this group of immunoceuticals based on their mode of action. The use of medicinal mushrooms to fight cancer has been well adopted in Korea, China, Japan, Russia, USA, and Canada. There is even an old Japanese legend that wild monkeys rarely experienced cancer, high blood pressure or diabetes, because they consumed wild mushrooms. Mushrooms of the family Polyporaceae are effective against esophageal, stomach, prostate and lung cancers. In 1957, Byerrum [48], for the first time confirmed the bioactivity of Basidiomycetes mushrooms and then isolated a *Boletus edulis* substance which could inhibit tumor cells from sarcoma S-180 [2]. Yohida et al. [49] isolated an active agent from *Lampteromyces japonicus* which worked against mouse Ehrlich carcinoma. Gregory (1966) experimented with more than 7000 cultures of mushrooms for antitumor activity against rodent tumors. Multiple inhibitory effects have been reported against sarcoma 180, mammary adenocarcinoma 755 and leukemia L-1210. Ikegawa et al. [50] reported antitumor activity against grafted sarcoma 180 in animals. Daba (1998) and Daba et al. [51] reported that *Pleurotus ostreatus* mushrooms grown on date wastes exhibited antitumor activity against Ehrlich ascites carcinoma. The antitumor activity was proved to be owing to a β-D-glucan mushroom polysaccharide (Mizuno, 1999).

In the late 1970s and 1980s, three anticancer polysaccharides, i.e., lentinan, schizophyllan and protein-bound polysaccharide (PSK, Krestin), were isolated from *Lentinus edodes*, *Schizophyllum commune* and *Coriolus versicolor*, respectively, and expanded in Japan [52]. Lentinan and schizophyllan are pure β-glucans [53,54,55], whereas PSK are protein-bound β-glucans [56,57]. A polysaccharopeptide (PSP) from *Coriolus versicolor* in China was also reported to be an anticancer and immunomodulatory agent [58].

Six mushroom polysaccharides have been investigated for their anticarcinogenic effect against human cancers, namely Lentinan, Schizophyllan, Maitake D-fraction, Active hexose correlated compounds (AHCC), polysaccharide-K and Polysaccharide-P. Lentinan is the polysaccharide that is most promising in this regard. It is produced from *Lentinus edodes,* commonly known as shiitake mushroom [59]. Lentinan is an isolated component of *Lentinus edodes* (shiitake). Countless studies on the anticancer effect of lentinan in animal and human carcinomas have been conducted. It was first isolated and studied by Chihara et al. [53], who validated that its anticarcinogenic effects were significantly greater than those of other mushroom polysaccharides. Maeda et al. [60], however, narrowed down the activity of lentinan for only specific types of tumors. Now, Lentinan is clinically used in the treatment of cancer in both China and Japan. As a drug, lentinan can be orally and intravenously administered. Generally, 1–2 mg lentinan is recommended for intravenous infusion. It is commonly used in bowel, liver, stomach, ovary, and lung cancer therapy in combination with other traditional pharmaceutical drugs. It is reported to increase the efficacy of treatment and therefore the survival of patients [61]. Natural compounds are able to successfully affect cellular proliferation, differentiation, apoptosis, angiogenesis, and metastasis [62]. Mushroom polysaccharides impact various types of cancers [59]. Possible mechanisms of anticarcinogenic activity of mushroom polysaccharides include the inhibition of tumor cell growth, apoptosis induction and immune stimulation.

Palomares et al. proved that the consumption of fresh/dried mushroom powder in pre- and post-menopausal females prevents breast cancer [63]. A novel macromolecular polysaccharide VGPI-a was purified from the fruiting bodies of the mushroom *Volvariella volvacea* using extraction methods such as ultrasound-assisted extraction, ion exchange and gel chromatography. VGPI-a is an α- glucan with a Mw of 1435.6 kDa and with a 1,4-linked D-Glcp backbone substituted at C-6 with 1-linked D-Glcp residue. VGPI-a displayed zero cytotoxicity on the macrophage RAW264.7 cells in vitro [64]; however, in terms of anticancer effect, it had a significant impact via enhancing the production and mRNA expression of NO, TNF-α, IL-6 and IL-1β in a dose-dependent manner. VGPI-a has also been documented to activate the MAPK signaling pathway by improving the phosphorylated levels of p38, JNK and ERK in RAW264.7 cells to promote the expression and secretion of the above cytokines.

In another study, the PAP-1a polysaccharides of *Pholiota adiposa* were isolated and the HPLGPC results revealed a 16.453 kDa PAP-1a made up of mannose, ribose, rhamnose, glucuronic acid, galacturonic acid, glucose, galactose, xylose, arabinose, and fucose. PAP-1a activated macrophages to secrete NO and cytokines such as TNF-a, IL-6, and IL-12p70. PAP-1a also inhibited Hep-G2, Hep-3B, and Huh7 via immunoregulation and triggered cell apoptosis by blocking the cell cycle in the G0/G1 phase. PAP80-2a, purified from *Pholiota adiposa* mycelia, is another type of polysaccharide also known for its anticancer applications [65].

A wide range of antitumor or immunostimulating polysaccharides with different chemical structures have been reported [8]. Some correlation has been established between the chemical structure and antitumor activities of mushroom polysaccharides. Homopolymers to highly complex heteropolymers [66] exhibit anticarcinogenic activity. Differences in the anticarcinogenic activity of polysaccharides have been related to their ability to solubilize in water, the size of the molecules, branching rate and form. It is reported that additional structural features such as β-(1-3) linkages in the backbone (main chain) of the glucan and additional β-(1-6)-branch points add up to the anticarcinogenic effect. β-glucans with only (1-6) glycosidic linkages have little or no activity. Higher molecular weight glucans have been reported by Mizuno et al. [67] and Mizuno [68] to be more effective than those of low molecular weight against cancers. Given this fact, it is true that mushroom polysaccharides vary in their chemical composition, structure and anticancer activity [69,70]. The anticancer, antioxidative, immunomodulating activities of *Ganoderma lucidum* [70] fruiting bodies, which contain the heteroglycan Glycopeptide, have been reported; and the anticancer and immunomodulating properties of *Lentinus edodes* [69] fruiting bodies, which contain glucan, are well established. *Pleurotus tuber-regium* [71] fruiting bodies contain β-D-glucan, which displays anticancer as well as hepatoprotective properties.

**Figure 3:**
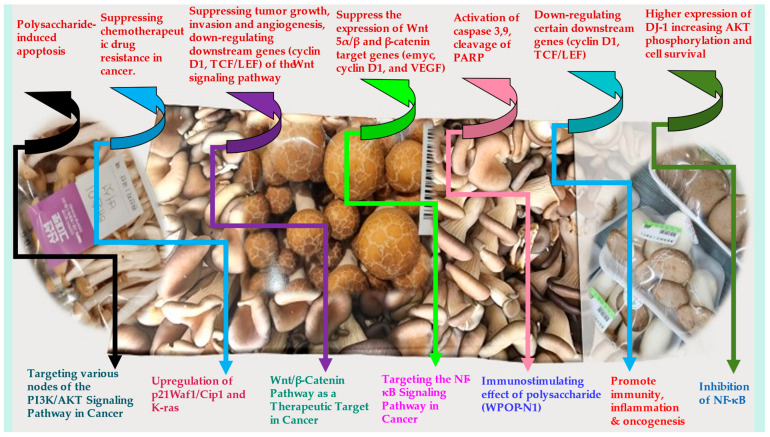
Overview of the anticancer mechanisms of mushroom/mushroom polysaccharides. Abbreviations: PI3Ks—phosphoinositide 3-kinases; AKT—serine/threonine protein kinase; TCF/LEF—T cell factor/lymphoid enhancer factor family; Wnt—wingless and Int-1; VEGF—vascular endothelial growth factor); PARP—poly adenosine diphosphate-ribose polymerase; DJ-1—Parkinson disease protein 7; p21Waf1/Cip1—cyclin-dependent Kinase Inhibitor; K-ras—Kirsten rat sarcoma viral oncogene homologue; NF-κB—Nuclear factor kappa B; WPOP-N1—Pleurotus ostreatus polysaccharide.

Several mushroom-derived components have shown direct antitumor activity and prevent oncogenesis and metastasis. Polysaccharides improve cancer-related symptoms when used in combination with chemotherapy. These induce the gene expression of several immunomodulating cytokines and their receptors. β-glucan, a mushroom-derived glucose polymer, stimulates NK cells, neutrophils, monocytes, macrophages, and T cells, as well as manifests immunomodulatory and antiproliferative effects. Schizophyllan, a β-D-glucan isolated from *Schizophyllum commune*, when used along with tamoxifen, decreased the breast tumor and initiated apoptosis in hepatic carcinomas. Figure 3 gives an overview of the anticancer mechanisms operating via mushroom bioactive extracts.

## 4. Clinical Trials Based on Mushroom Polysaccharides

Mushroom polysaccharides, especially ß-glucans, krestin and polysaccharide peptide (PSP) from *Coriolus versicolor* and lentinan, isolated from *Lentinula edodes* (shiitake), have been well studied in humans [72]. Clinical studies conducted on the complementary use of mushroom polysaccharides combined with chemotherapy have led to the disease-free survival of colorectal cancer patients and improved the quality of life among lung cancer patients [73,74]. Lentinan has successfully prolonged the overall survival of gastric and colorectal carcinomas in cancer patients [75,76,77]. Recurrent gastric cancer patients showed prolonged median survival rates. In a randomized controlled study, the tegafur/combination of lentinan and tegafur significantly prolonged the overall survival rates. In Japan, during cancer chemotherapy on solid tumors, the patients administered lentinan had a significantly higher response rate (14.9%) than the patients without. The use of lentinan in conjunction with other chemotherapeutic agents decreased the side-effects of chemotherapy, such as nausea, pain, hair loss and lowered immune status. Lentinan is now clinically applied for cancer treatment in both China and Japan. Lentinan can be both orally and intravenously administered in dosages of 1–2 mg as recommended by the Chinese Food and Drug Administration (SFDA) for intravenous infusion. Although the 123 independent studies show varied response rates, all chemotherapy plus lentinan groups showed promising results [78].

A large number of clinical trials have been demonstrated in Japan and schizophyllan has been approved for clinical use. Clinical studies using schizophyllan combined with conventional chemotherapy (tegafur or mitomycin C and 5-fluorouracil) was applied to 367 patients with recurrent and inoperable gastric cancer, and an increase in the survival rates was evident [79], although this was inconsistent [80]. Schizophyllan has been reported to improve the overall survival rates of head and neck-related cancer cases [81]. In another randomized controlled study of schizophyllan in combination with radiotherapy, schizophyllan consistently improved the overall survival of stage II cervical cancer patients; this, however, was not the case with respect to stage III patients [82,83]. Another randomized clinical trial involving 312 patients, following surgery, radiotherapy, chemotherapy (fluorouracil) and schizophyllan in various combinations, showed positive results [84].

Kamiyama [85] conducted a clinical trial in Japan to evaluate the preventive effect of active hexose-correlated compound (AHCC) against the recurrence of hepatocellular carcinoma following surgical resection. Their results showed that of the 300 cancer patients administered AHCC, 58 were effectively treated, and 46 showed complete or partial regression. Jones [86] reported an early pilot study from China involving 63 cancer patients against solid tumor, which showed a success rate of nearly 95% and 90% for leukemia.

Nanba [87,88] observed the tumor regression/remarkable symptomatic improvement in 11 out of 15 hepatocellular carcinomas with D-fraction plus Maitake. A combination of D-fraction plus Maitake showed an increase of 12–28%. The Food and Drug Administration (USA) approved Grifon-D (GD) for clinical trials in patients with advanced cancer, and various other US-based clinical trials using mushroom polysaccharides are also underway at various institutions [87].

Deng et al. [89] recorded the response to the oral intake of *G. frondosa* polysaccharide extracts in 34 postmenopausal breast cancer patients. They reported that these patients became disease-free after primary treatment as part of a phase I/II trial. They observed marked increases in TNF-α, IL-2, and IL-10 production and a one-fifth reduction in IFN-γ production. Grinde et al. [90] reported positive changes in mRNA (qPCR), in a clinical trial with chronic hepatitis patients when β-glucan mushroom polysaccharides from *Agaricus blazei* were used.

Clinical trials have been performed on the following medicinal mushrooms (MMs): *Agaricus bisporus* (single trial, [91]); *A. blazei* (three trials, [90,92,93]); *A. sylvaticus* (two trials; [94,95]); *Antrodia cinnamomea* (single trial, [44]); *Coriolus versicolor* (two trials, [36,37]); *Ganoderma lucidum* (single trial, [35]); *Grifola frondosa* (three trials, [89,96,97]); *Lentinus edodes* (four trials, [98,99,100,101]); *Phellinus rimosus* (single trial, [102]); and *Poria cocos* (single trial, [103]).

AndosanTM (ACE Co. Ltd. produced for Immunopharma, Gifu-ken, Japan, is a product made from the mycelium of *Agaricus blazei,* as well as smaller amounts of *Grifola frondosa* (3%) and *Hericium erinaceus* (15%). This has been successfully tested in various clinical trials, demonstrating anticancer, anti-inflammatory, and antiallergic action, because of the mushroom-derived β-glucans and isoflavonoids [104]. It is said that the β-glucana stimulate the Peyer’s patches in the gut-associated lymphoid tissue (GALT), together with other less-defined absorbable low-molecular-weight (LMW) substances, such as flavonoids [104]. In 2015, Tangen et al. [93] reported the oral administration of Andosan (60 mL/d) for seven weeks to patients with multiple myeloma undergoing high-dose chemotherapy with autologous stem cell transplantation (ASCT). Increased CD4+, CD127d+, and CD25+ Treg cells and plasmacytoid dendritic cells (CD303+) were observed. Moreover, significant increases in serum levels of interleukins IL-1ra (receptor antagonist), IL-5, and IL-7 were also reported [105]. Table 2 highlights the milestones achieved by mushroom polysaccharides in cancer mycotherapy.

## 5. Challenges and Future Perspectives

Mushroom polysaccharides and their positive effects in terms of their contributions to anticancer activity have been reviewed and confirmed as consistently valid. However, on the flip side, the adverse events (AE) following treatment with mushroom polysaccharides have not been that well documented. Few clinical trials have described the adverse effects of mushroom extracts [35,44,106]. Breast cancer patients undergoing onendocrine therapy along with *G. lucidum* recorded mild discomfort such as dizziness and dry mouth [35]. The mycelial extracts of Lentinula failed to reduce prostate-specific antigen levels in a phase II study of 74 prostate cancer patients [100]. Additionally, White et al. [99] recorded the inability of shiitake mushroom extract to lower the prostate-specific antigen levels or even keep them stable in 62 prostate cancer patients. Such adverse/ill effects/nil effects have not been determined in the case of mushroom polysaccharides.

There are other reports that confirm the inability and incapacity of mushroom extracts to treat cancer. Fortes et al. [107] followed 56 post-surgery colorectal cancer patients treated with *Agaricus sylvaticus* extract with no improvement in their quality of life [107]. In another clinical trial with 37 patients with lung, breast, liver, stomach, and colorectal advanced adenocarcinoma who were undergoing chemotherapy for 30 d, when *Antrodia cinnamomea* was administered, no significant improvements other than sleep (*p* = 0.04) [44] were recorded. More frequent but less intense (grade 1 and 2) gastrointestinal symptoms (abdominal pain and diarrhea) were reported. Oka et al. [106] reported diarrhea, stomach discomfort and poor health in 6 out of 123 colorectal adenoma cases receiving *G. lucidum*. Such adverse effects or inabilities of mushroom polysaccharides have not been ideally worked out. This review emphasizes that this gap needs to be filled so that the holistic clinical potential of mushroom polysaccharides can be worked out and therapies improved upon.

Few clinical studies have been conducted with mushroom polysaccharides, however, they have all been conducted within a limited framework. The clinical testing protocols are still rather unstructured, having dominant shortcomings and gaps. No standard procedure for the evaluation of results is available, and the value of such studies is undermined in several respects [108,109]. Moreover, not all trials were randomized or had a placebo control [110,111] nor were double-blinded where safety and side effects are neglected. Some studies also rely on subjective assessments such as the quality of life so that the examined result may not have any real scientific value [92,112]. Again, in most cases, the so-called pilot or phase I studies have no follow-up. The results thus remain only wholly preliminary.

It is also difficult to compare the results obtained in separate clinical studies. This is because the preparation method used to carry out the trial plays a crucial role. Even for the same fungal species tested opposite, different results were obtained, as in the case of Yoshimura et al. [113] and Ohno et al. [112] with *A. blazei* Murrill. This is because the extraction procedures were not clearly standardized. Similarly, in another case, whatever was tested for a specific action has already been proven in other trials, and may become completely ineffective, or deleterious, worsening the clinical picture [114,115]. This is because of the extraction procedure, concentration of the metabolite and the age, as well as the location-related influences of the mushroom batch used. Additionally, under similar conditions, the medicinal properties can vary enormously depending on the strain, the geographical area, the growing conditions and the substrate used, the part of the mushroom used, and the growing stage at the moment of processing. All these parameters changed the composition of the mushroom and, consequently, its bioactive capacity. Thus, since these aspects are case sensitive, there is no way that the results can be generalized. This has to be worked on and working procedures must be optimized.

In recent years, research into medicinal mushrooms has progressed exponentially, but is far from over. Many species are yet to be explored for their pharmacological properties. The identification of the anticancer biomolecules from various mushroom extracts is lacking. The metabolites responsible for the anticancer activity, their chemical characterization, and their mechanism of action have been under-investigated. There is also an urgent need to know their individual and synergistic effects and their in vivo dynamics. It is also necessary to standardize the production of mushroom supplements throughout the supply chain, from cultivation to extraction and the preparation of the commercial formulation, as well as precise monitoring and regulation to ensure high quality levels and yield consistent results.

Several studies have been conducted; however, with the promising potential of mushroom polysaccharides, there is a long way to go. Mushroom polysaccharides could have a lot more to offer, which needs to be disclosed. Additionally, it was seen that only the same few mushroom types have been worked upon. With exhaustive list of edible mushrooms, it is crucial that the work on mushroom polysaccharides be expanded. The other concern includes lapses in the proper utilization of mushroom polysaccharides for anticancer activity [105].

**Figure 4:**
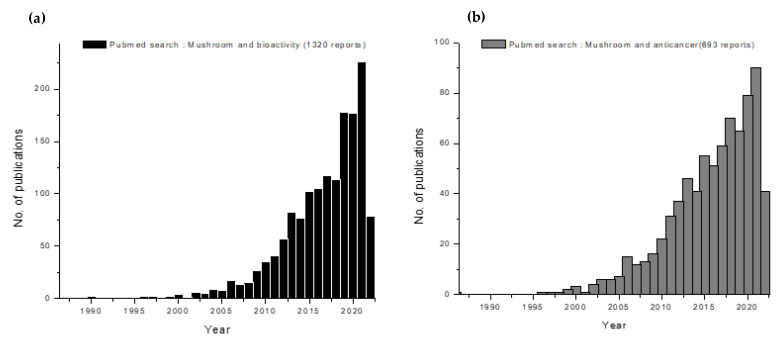

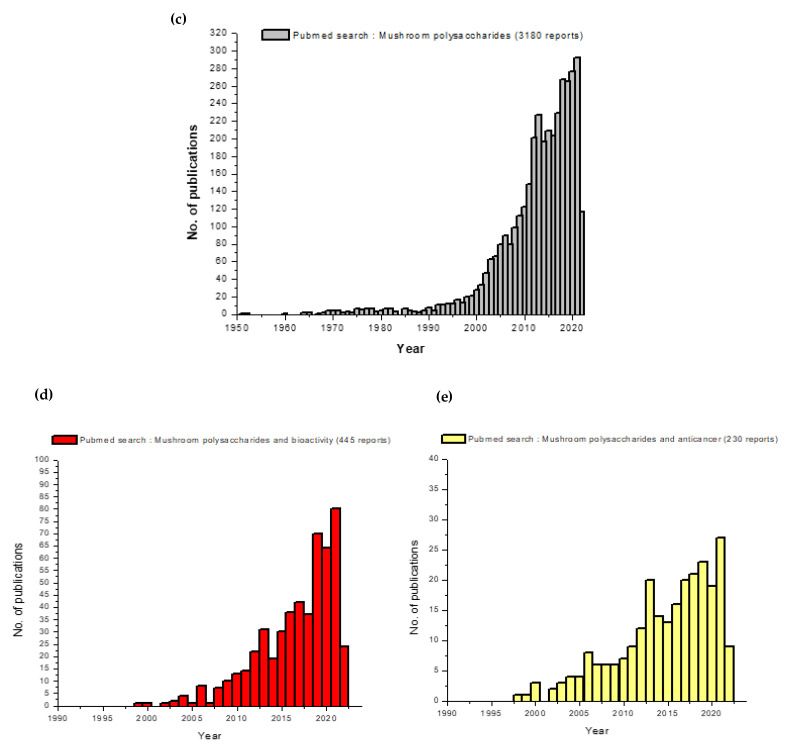
A pubmed-based search showing the updated trend in medicinal mushroom research. The keywords that defined the search were (**a**) mushroom and bioactivity (1320 reports); (**b**) mushroom and anticancer (693 reports); (**c**) mushroom polysaccharides (3180 reports); (**d**) mushroom polysaccharides and bioactivity (445 reports); and (**e**) mushroom polysaccharides and anticancer (230 reports).

Figure 4 highlights the fact that mushroom polysaccharides with respect to anticancer applications need to be worked out more elaborately. As highlighted by our pubmed search using mushroom-related keywords such as: ‘mushroom and bioactivity’, which were backed up by 1320 reports (Figure 4a); ‘mushroom and antitumor’ (693 reports) (Figure 4b); ‘mushroom polysaccharides’ (3180 reports) (Figure 4c); ‘mushroom polysaccharides and bioactivity’ (445 reports) (Figure 4d); and ‘mushroom polysaccharides and anticancer’ (230 reports) (Figure 4e). This keyword search clearly indicates that the application of mushroom polysaccharides in anticancer studies has been much less researched than mushroom extracts versus anticancer activity. There is room for expanding and extrapolating mushroom polysaccharides to cancer mycotherapy with more fervor.

## 6. Conclusions

The benefits of mushroom polysaccharides and the milestones reached with respect to anticancer activity have been reviewed. The successful use of mushroom polysaccharides for clinical investigations and the progress made in the application area of clinical trials have been summarized. The challenges facing the use of mushroom polysaccharides as well as their limitations were also discussed under a future prospective. The need to explore and standardize has been emphasized. Optimization is crucial for the translation of the available bioactivity to clinical studies. More human trials and clinical experiments are needed. The commercialization of trustworthy and tested well-accomplished mushroom products should be a potentially rewarding area, if focused appropriately.

## Figures and Tables

**Table 1:** Consolidated list of anticarcinogenic mushrooms.

Mushroom	Bioactive Component for Anticancer Activity	Anticancer Application	Reference
*Albatrellus confluens*	Grifolin and Neogrifolin	Against osteosarcoma U2OS, MG63 cell line lines	[11]
*Auricularia auricula*	Polysaccharide	Against liver cancer	[12]
*Grifola frondosa*	Polysaccharide	Against liver and breast cancer	[13]
*Lentinus crinitus*	Panepoxydone (PP)	Against breast cancer	[14]
*Lentinula edodes*	Protein latcripin-1,3,13,15	Against lung cancer	[15]
*Lentinus edodes*	Polysaccharide	Against hepatocarcinoma of mouse	[16,17,18,19]
*Pleurotus ferulae*	Terpenoids	Against melanoma/gastric cancer	[20]
*Pleurotus ostreatu*s	Polysaccharide	Against sarcoma cells	[21]
*Termitomyces clypeatus*	Sugar entities	Against brain, breast, acute myeloid leukemia, lung, ovary, retinoblastoma	[22]
*Amauroderma rude*	Ergosterol	Against breast cancer	[23]
*Antrodia camphorata*	Polysaccharide (ACE)	Against hepatocellular carcinoma	[24]
*Antrodia camphorata*	Antroquinonol	Against pancreatic carcinoma colon cancer	[25]
*Antrodia camphorata*	4-Acetylantroquinonol B	Against colorectal cancer	[26]
*Cordyceps militaris*	Cordycepin	Against NRK-52E	[27]
*Fomes fomentarius*	Polysaccharide (MFKF-AP1β)	Against lung cancer	[28]
*Grifola frondosa*	Sulfated polysaccharides	Against liver cancer	[29],
*G lucidum*	Polysaccharide	Against liver cancer	[12]
*Inonotus obliquus*	Ergosterol peroxide	Against colorectal cancer	[30]
*Phellinus linteus*	Protein-bound polysaccharide	Against colon cancer	[31]
*Phellinus linteus*	Hispolon	Against human hepatoma	[32]
*Phellinus linteus*	Polysaccharide	Against liver cancer	[33]

**Table 2:** Clinical studies based on mushroom polysaccharides-based mycotherapy.

Mushroom sps	Type of Cancer	Polysaccharide	Details of Clinical Study
*Agaricus bisporus*	Ovarian	Mushroom polysaccharide	Correlated mushroom intake and epithelial ovarian cancer in 500 participants, an observational study
*Andosan^TM^, a product made from the mycelium of Agaricus blazei, as well as smaller amounts of Grifola frondosa (3%) and Hericium erinaceus (15%),*	Various cancers	β-glucans	Tested in various clinical trials, demonstrating antitumor, anti-inflammatory, and antiallergic action
*Andosan^TM^, a product made from the mycelium of Agaricus blazei, as well as smaller amounts of Grifola frondosa (3%) and Hericium erinaceus (15%),*	Multiple myeloma undergoing high-dose chemotherapy with autologous stem cell transplantation (ASCT)	β-glucans	Andosan^TM^ extract was orally administered (60 mL/d) for seven weeks to patients, the overall survival increased notably
*Lentinula edodes*	Esophageal cancer	Lentinan	Lentinan + RT treated, decrease in RT toxicity
*Lentinula edodes*	Gastric cancer	Lentinan	Lentinan + XELOX chemotherapy; enhanced XELOX chemotherapy response rate and performance status, decreased CT toxicity
*Lentinula edodes*	Unresectable/recurrent gastric cancer	Lentinan	Lentinan + CT the MST was 139 days and CT alone given then MST was 114 days; increase in survival rate and response rate
*Lentinula edodes*	Colorectal cancer	Lentinan	Lentinan + FOLFOX chemotherapy combined gave better response rate, performance status, and decreased CT toxicity
*Lentinula edodes*	Advanced (gastrointestinal, liver and lung) cancer	Lentinan	Lentinan + CT; enhanced survival rate, response rate, PoD, decreased CT toxicity
*Lentinula edodes*	Lung cancer	Lentinan	Lentinan + CT combined the resp. rate was 56.9% while for CT alone the resp. rate was 43.3%; lentinan led to higher response rate
*Lentinula edodes*	Non-small cell lung cancer	Lentinan	Lentinan + CT -better response rate, decreased CT toxicity
*Lentinula edodes*	Malignant pleural effusion	Lentinan	Lentinan (intrapleural infusion) + CT led to enhanced response rate and QoL, decreased CT toxicity
*Coriolus versicolor*	Gastric, oesophageal, colorectal, breast and lung cancers	Protein bound polysaccharide PSK, Krestin	Immunostimulant, inhibits tumor growth Orally administered
*Coriolus versicolor*	Gastric, esophageal, colorectal, breast and lung cancers	Polysaccharide peptide PSP	Immunostimulant, inhibit tumor growth Oral administration
*Grifola frondosa*	Lung, lingual, breast, gastric, or liver cancer	D-fraction (β-glucan)	Inhibition of the progression of metastasis and reduced expression of (carcinoembryonic antigen (CEA) and cancer antigen 15–3 (CA15–3) and CA19–9) tumor markers
*Schizophyllum commune*	Gastric, cervical cancer	Polysaccharide SPG, Sonifilan	Immunostimulant, intratumorally administered
*Schizophyllum commune*	Recurrent and inoperable gastric cancer	Schizophyllan combined with conventional chemotherapy (tegafur or mitomycin C and 5-fluorouracil	367 patients studied with positive results
*Schizophyllum commune*	Cervical cancer	Schizophyllan in combination with radiotherapy,	Schizophyllan consistently improved the overall survival of stage II cervical cancer patients
*Grifola frondosa*	Hepatocellular carcinomas	D-fraction plus Maitake	11 out of 15 hepatocellular carcinomas treated with a combination of D-fraction plus Maitake, showed an increased overall rate of 12–28%
*Ganoderma lucidum*	Lung cancer	Ganopoly^®^ (a polysaccharide product from *G. lucidum*	Randomized, double-blind, placebo-controlled, multicenter clinical trial was performed in 68 patients; improvement in immunological functions, with significant increases in plasma IL-2, IL-6, and IFN-γ concentrations, Cd56+, phytohemagglutinin (PHA) responses and NK activity; significant decreases in IL-1 and TNF-α

Legend: CT = chemotherapy; MST = median survival time; PoD = progression of disease; RT = radiotherapy.

## Data Availability

Not applicable.

## References

[1] Chang S.-T., Miles P.G. (2008). Mushrooms: Cultivation, Nutritional Value, Medicinal Effect, and Environmental Impact.

[2] Patel S., Goyal A. (2012). Recent developments in mushrooms as anti-cancer therapeutics: A review. 3 Biotech.

[3] Chang S.T., Wasser S.P. (2012). The role of culinary-medicinal mushrooms on human welfare with a pyramid model for human health. Int. J. Med. Mushrooms.

[4] Finimundy T.C., Gambato G., Fontana R., Camassola M., Salvador M., Moura S., Hess J., Henriques J.A., Dillon A.J., Roesch-Ely M. (2013). Aqueous extracts of *Lentinula edodes* and *Pleurotus sajor-caju* exhibit high antioxidant capability and promising in vitro antitumor activity. Nutr. Res..

[5] Yu S., Weaver V., Martin K., Cantorna M.T. (2009). The effects of whole mushrooms during inflammation. BMC Immunol..

[6] Zhang L.X., Fan C., Liu S.C., Zang Z.F., Jiao L.L., Zhang L.P. (2011). Chemical composition and antitumor activity of polysaccharide from *Inonotus obliquus*. J. Med. Plants Res..

[7] Chen J.Z., Seviour R. (2007). Medicinal importance of fungal beta-(1 -> 3), (1 -> 6)-glucans. Mycol. Res..

[8] Wasser S.P. (2002). Medicinal mushrooms as a source of antitumor and immunomodulating polysaccharides. Appl. Microbiol. Biotechnol..

[9] Yap A.T., Ng M.L.M. (2001). An improved method for the isolation of Lentinan from the edible and Medicinal Shiitake mushroom, *Lentinus edodes* (Burk) Sing (*Agaricomycetideae*). Int. J. Med. Mushroom.

[10] Kumar K., Mehra R., Guine R.P.F., Lima M.J., Kumar N., Kaushik R., Ahmed N., Yadav A.N., Kumar H. (2021). Edible Mushrooms: A Comprehensive Review on Bioactive Compounds with Health Benefits and Processing Aspects. Foods.

[11] Jin S., Pang R.P., Shen J.N., Huang G., Wang J., Zhou J.G. (2007). Grifolin induces apoptosis via inhibition of PI3K/AKT signalling pathway in human osteosarcoma cells. Apoptosis.

[12] OuYang F., Wang G., Guo W., Zhang Y., Xiang W., Zhao M. (2013). AKT signalling and mitochondrial pathways are involved in mushroom polysaccharide-induced apoptosis and G1 or S phase arrest in human hepatoma cells. Food Chem..

[13] Lin C.H., Chang C.Y., Lee K.R., Lin H.J., Lin W.C., Chen T.H., Wan L. (2016). Cold-water extracts of *Grifola frondosa* and its purified active fraction inhibit hepatocellular carcinoma in vitro and in vivo. Exp. Biol. Med..

[14] Arora R., Yates C., Gary B.D., McClellan S., Tan M., Xi Y., Reed E., Piazza G.A., Owen L.B., Dean-Colomb W. (2014). Panepoxydone targets NF-kB and FOXM1 to inhibit proliferation, induce apoptosis and reverse epithelial to mesenchymal transition in breast cancer. PLoS ONE.

[15] Liu B., Zhong M., Lun Y., Wang X., Sun W., Li X., Ning A., Cao J., Zhang W., Liu L. (2012). A novel apoptosis correlated molecule: Expression and characterization of protein Latcripin-1 from *Lentinula edodes* C(91-3). Int. J. Mol. Sci..

[16] You R.X., Liu J.Y., Li S.J., Wang L., Wang K.P., Zhang Y. (2014). Alkali-soluble polysaccharide, isolated from *Lentinus edodes*, induces apoptosis and G2/M cell cycle arrest in H22 cells through microtubule depolymerization. Phytother. Res..

[17] Tian L., Wang X., Li X., Liu B., Zhang W., Cao J., Ning A., Huang M., Zhong M. (2016). In vitro antitumor activity of Latcripin-15 regulator of chromosome condensation 1 domain protein. Oncol. Lett..

[18] Wang J., Zhong M., Liu B., Sha L., Lun Y., Zhang W., Li X., Wang X., Cao J., Ning A. (2015). Expression and functional analysis of novel molecule—Latcripin-13 domain from *Lentinula edodes* C91-3 produced in prokaryotic expression system. Gene.

[19] Ann X.H., Lun Y.Z., Zhang W., Liu B., Li X.Y., Zhong M.T., Wang X.L., Cao J., Ning A.H., Huang M. (2014). Expression and characterization of protein Latcripin-3, an antioxidant and antitumor molecule from *Lentinula edodes* C91-3. Asian Pac. J. Cancer Prev..

[20] Wang W., Chen K., Liu Q., Johnston N., Ma Z., Zhang F., Zheng X. (2014). Suppression of tumor growth by *Pleurotus ferulae* ethanol extract through induction of cell apoptosis, and inhibition of cell proliferation and migration. PLoS ONE.

[21] Kong F., Li F.E., He Z., Jiang Y., Hao R., Sun X., Tong H. (2014). Anti-tumor and macrophage activation induced by alkali-extracted polysaccharide from *Pleurotus ostreatus*. Int. J. Biol. Macromol..

[22] Mondal A., Banerjee D., Majumder R., Maity T.K., Khowala S. (2016). Evaluation of in vitro antioxidant, anticancer and in vivo antitumour activity of Termitomyces clypeatus MTCC 5091. Pharm. Biol..

[23] Li X., Wu Q., Xie Y., Ding Y., Du W.W., Sdiri M., Yang B.B. (2015). Ergosterol purified from medicinal mushroom Amauroderma rude inhibits cancer growth in vitro and in vivo by up-regulating multiple tumor suppressors. Oncotarget.

[24] Chang J.S., Kuo H.P., Chang K.L., Kong Z.L. (2015). Apoptosis of Hepatocellular Carcinoma Cells Induced by Nanoencapsulated Polysaccharides Extracted from *Antrodia camphorata*. PLoS ONE.

[25] Yu C.C., Chiang P.C., Lu P.H., Kuo M.T., Wen W.C., Chen P., Guh J.H. (2012). Antroquinonol, a natural ubiquinone derivative, induces a cross talk between apoptosis, autophagy and senescence in human pancreatic carcinoma cells. J. Nutr. Biochem..

[26] Chang T.C., Yeh C.T., Adebayo B.O., Lin Y.C., Deng L., Rao Y.K., Huang C.C., Lee W.H., Wu A.T., Hsiao M. (2015). 4-Acetylantroquinonol B inhibits colorectal cancer tumorigenesis and suppresses cancer stem-like phenotype. Toxicol. Appl. Pharmacol..

[27] Kadomatsu M., Nakajima S., Kato H., Gu L., Chi Y., Yao J., Kitamura M. (2012). Cordycepin as a sensitizer to tumour necrosis factor (TNF)-alpha-induced apoptosis through eukaryotic translation initiation factor 2 alpha (eIF2 alpha)- and mammalian target of rapamycin complex 1 (mTORC1)-mediated inhibition of nuclear factor (NF)-kappa beta. Clin. Exp. Immunol..

[28] Kim S.H., Jakhar R., Kang S.C. (2015). Apoptotic properties of polysaccharide isolated from fruiting bodies of medicinal mushroom Fomes fomentarius in human lung carcinoma cell line. Saudi J. Biol. Sci..

[29] Wang C.L., Meng M., Liu S.B., Wang L.R., Hou L.H., Cao X.H. (2013). A chemically sulfated polysaccharide from *Grifola frondos* induces HepG2 cell apoptosis by notch1-NF-kappa B pathway. Carbohydr. Polym..

[30] Kang J.H., Jang J.E., Mishra S.K., Lee H.J., Nho C.W., Shin D., Jin M., Kim M.K., Choi C., Oh S.H. (2015). Ergosterol peroxide from Chaga mushroom (*Inonotus obliquus*) exhibits anti-cancer activity by down-regulation of the beta-catenin pathway in colorectal cancer. J. Ethnopharmacol..

[31] Song K.S., Li G., Kim J.S., Jing K., Kim T.D., Kim J.P., Seo S.B., Yoo J.K., Park H.D., Hwang B.D. (2011). Protein-bound polysaccharide from *Phellinus linteus* inhibits tumor growth, invasion, and angiogenesis and alters Wnt/beta-catenin in SW480 human colon cancer cells. BMC Cancer.

[32] Huang G.J., Yang C.M., Chang Y.S., Amagaya S., Wang H.C., Hou W.C., Huang S.S., Hu M.L. (2010). Hispolon Suppresses SK-Hep1 Human Hepatoma Cell Metastasis by Inhibiting Matrix Metalloproteinase-2/9 and Urokinase-Plasminogen Activator through the PI3K/Akt and ERK Signaling Pathways. J. Agric. Food Chem..

[33] Xu W.W., Huang J.J.H., Cheung P.C.K. (2012). Extract of *Pleurotus pulmonarius* Suppresses Liver Cancer Development and Progression through Inhibition of VEGF-Induced PI3K/AKT Signaling Pathway. PLoS ONE.

[34] Chun S., Gopal J., Muthu M. (2021). Antioxidant Activity of Mushroom Extracts/Polysaccharides-Their Antiviral Properties and Plausible AntiCOVID-19 Properties. Antioxidants.

[35] Zhao H., Zhang Q., Zhao L., Huang X., Wang J., Kang X. (2012). Spore Powder of Ganoderma lucidum Improves Cancer-Related Fatigue in Breast Cancer Patients Undergoing Endocrine Therapy: A Pilot Clinical Trial. Evid. Based Complement. Alternat. Med..

[36] Torkelson C.J., Sweet E., Martzen M.R., Sasagawa M., Wenner C.A., Gay J., Putiri A., Standish L.J. (2012). Phase 1 Clinical Trial of Trametes versicolor in Women with Breast Cancer. ISRN Oncol..

[37] Chay W.Y., Tham C.K., Toh H.C., Lim H.Y., Tan C.K., Lim C., Wang W.W., Choo S.P. (2017). Coriolus versicolor (Yunzhi) Use as Therapy in Advanced Hepatocellular Carcinoma Patients with Poor Liver Function or Who Are Unfit for Standard Therapy. J. Altern. Complement. Med..

[38] Wang Z.J., Luo D.H., Liang Z.Y. (2004). Structure of polysaccharides from the fruiting body of *Hericium erinaceus* Pers. Carbohydr. Polym..

[39] Synytsya A., Mickova K., Synytsya A., Jablonsky I., Spevacek J., Erban V., Kovarikova E., Copikova J. (2009). Glucans from fruit bodies of cultivated mushrooms *Pleurotus ostreatus* and *Pleurotus eryngii*: Structure and potential prebiotic activity. Carbohydr. Polym..

[40] Sarikurkcu C., Tepe B., Yamac M. (2008). Evaluation of the antioxidant activity of four edible mushrooms from the Central Anatolia, Eskisehir-Turkey: *Lactarius deterrimus*, *Suillus collitinus*, *Boletus edulis*, *Xerocomus chrysenteron*. Bioresour. Technol..

[41] Kim H.G., Yoon D.H., Lee W.H., Han S.K., Shrestha B., Kim C.H., Lim M.H., Chang W., Lim S., Choi S. (2007). *Phellinus linteus* inhibits inflammatory mediators by suppressing redox-based NF-kappaB and MAPKs activation in lipopolysaccharide-induced RAW 264. 7 macrophage. J. Ethnopharmacol..

[42] Carneiro A.A., Ferreira I.C., Duenas M., Barros L., da Silva R., Gomes E., Santos-Buelga C. (2013). Chemical composition and antioxidant activity of dried powder formulations of *Agaricus blazei* and *Lentinus edodes*. Food Chem..

[43] Brown A.C., Waslien C.I., Finglas L.T.A.P.M. (2003). Stress and nutrition. Encyclopedia of Food Sciences and Nutrition.

[44] Tsai M.Y., Hung Y.C., Chen Y.H., Chen Y.H., Huang Y.C., Kao C.W., Su Y.L., Chiu H.H., Rau K.M. (2016). A preliminary randomised controlled study of short-term *Antrodia cinnamomea* treatment combined with chemotherapy for patients with advanced cancer. BMC Complement. Altern. Med..

[45] Lindequist U., Niedermeyer T.H.J., Julich W.D. (2005). The pharmacological potential of mushrooms. Evid. Based Complement. Altern..

[46] Wasser S.P., Weis A.L. (1999). Therapeutic effects of substances occurring in higher basidiomycetes mushrooms: A modern perspective. Crit. Rev. Immunol..

[47] Wasser S.P., Weis A. (1999). Medicinal properties of substances occurring in higher Basidiomycetes mushrooms: Current perspectives. Int. J. Med. Mushroom.

[48] Byerrum R.U., Clarke D.A., Lucas E.H., Ringler R.L., Stevens J.A., Stock C.C. (1957). Tumor inhibitors in *Boletus edulis* and other Holobasidiomycetes. Antibiot. Chemother..

[49] Yoshida T.O., Rising J.A., Nungester W.J. (1962). A tumor inhibitor in *Lampteromyces japonica*. Proc. Soc. Exp. Biol. Med..

[50] Ikekawa T., Nakanishi M., Uehara N., Chihara G., Fukuoka F. (1968). Antitumor Action of Some Basidiomycetes Especially *Phellinus linteus*. GANN.

[51] Daba A.S., Wissa Jwanny E., Esmat A.Y., Rashad M., Fattah A. (2002). Antitumor activity of polysaccharides from *Pleurotus ostreatus* fruiting bodies and my-celia cultivated on date waste media. Egypt. J. Biochem. Mol. Biol..

[52] Mizuno T., Saito H., Nishitoba T., Kawagishi H. (1995). Antitumor-Active Substances from Mushrooms. Food Rev. Int..

[53] Chihara G., Hamuro J., Maeda Y., Arai Y., Fukuoka F. (1970). Fractionation and Purification of Polysaccharides with Marked Antitumor Activity, Especially Lentinan, from *Lentinus-edodes* (Berk) Sing (an Edible-Mushroom). Cancer Res..

[54] Komatsu N., Okubo S., Kikumoto S., Kimura K., Saito G., Sakai S. (1969). Host-Mediated Antitumor Action of Schizophyllan a Glucan Produced by Schizophyllum Commune. GANN.

[55] Chihara G. (1992). Immunopharmacology of Lentinan, a polysaccharide isolated from *Lentinus edodes*: Its application as a host defence potentiator. Int. J. Orient. Med..

[56] Kobayashi H., Matsunaga K., Oguchi Y. (1995). Antimetastatic effects of PSK (Krestin), a protein-bound polysaccharide obtained from basidiomycetes: An overview. Cancer Epidemiol. Biomark. Prev..

[57] Tsukagoshi S., Hashimoto Y., Fujii G., Kobayashi H., Nomoto K., Orita K. (1984). Krestin (PSK). Cancer Treat. Rev..

[58] Yang Q.Y., Jong S.C., Li X.Y., Zhou J.X., Chen R.T., Xu L.Z. (1992). Antitumor and Immunomodulating Activities of the Polysaccharide-Peptide (Psp) of Coriolus-Versicolor. Eos-Riv. Immunol..

[59] Meng X., Liang H.B., Luo L.X. (2016). Antitumor polysaccharides from mushrooms: A review on the structural characteristics, antitumor mechanisms and immunomodulating activities. Carbohydr. Res..

[60] Maede Y.Y., Hamuro J., Chihara G. (1974). The nature of immunopotentiation by the antitumor polysaccharide Lentinan and the significance of biogenic amines in its action. Int. J. Cancer.

[61] Wu S.Y., Yan M.D., Wu A.T.H., Yuan K.S.P., Liu S.H. (2016). Brown Seaweed Fucoidan Inhibits Cancer Progression by Dual Regulation of mir-29c/ADAM12 and miR-17-5p/PTEN Axes in Human Breast Cancer Cells. J. Cancer.

[62] Mitra S., Dash R. (2018). Natural Products for the Management and Prevention of Breast Cancer. Evid. Based Complement. Altern..

[63] Palomares M.R., Rodriguez J., Phung S., Stanczyk F.Z., Lacey S.F., Synold T.W., Denison S., Frankel P.H., Chen S. (2011). A dose-finding clinical trial of mushroom powder in postmenopausal breast cancer survivors for secondary breast cancer prevention. J. Clin. Oncol..

[64] Cui F.J., Jiang L.H., Qian L.S., Sun W.J., Tao T.L., Zan X.Y., Yang Y., Wu D., Zhao X. (2020). A macromolecular alpha-glucan from fruiting bodies of Volvariella volvacea activating RAW264. 7 macrophages through MAPKs pathway. Carbohydr. Polym..

[65] Yang Z.W., Liu Z.J., Xu J., Zhu J.M., Pu Y.W., Bao Y.X. (2022). Study on the physicochemical properties and immunomodulatory anti-tumor effect of the Pholiota adiposa polysaccharide. Food Funct..

[66] Ooi V.E.C., Liu F. (1999). A review of pharmacological activities of mushroom polysaccharides. Int. J. Med. Mushrooms.

[67] Mizuno T., Yeohlui P., Kinoshita T., Zhuang C., Ito H., Mayuzumi Y. (1996). Antitumor activity and chemical modification of polysaccharides from *Niohshimeji* mushroom, *Tricholma giganteum*. Biosci. Biotechnol. Biochem..

[68] Mizuno T. (1999). The extraction and development of antitumor active polysaccharides from medicinal mushrooms in Japan. Int. J. Med. Mushrooms.

[69] Rincao V.P., Yamamoto K.A., Ricardo N.M.P.S., Soares S.A., Meirelles L.D.P., Nozawa C., Linhares R.E.C. (2012). Polysaccharide and extracts from *Lentinula edodes*: Structural features and antiviral activity. Virol. J..

[70] Miyazaki T., Nishijima M. (1981). A Novel Glycosaminoglycan from the Fungus *Omphalia lapidescence*. Carbohydr. Res..

[71] Staniszewska J., Szymański M., Ignatowicz E. (2017). Antitumor and immunomodulatory activity of *Inonotus obliquus*. Herba Pol..

[72] Joseph T.P., Chanda W., Padhiar A.A., Batool S., LiQun S., Zhong M., Huang M. (2018). A Preclinical Evaluation of the Antitumor Activities of Edible and Medicinal Mushrooms: A Molecular Insight. Integr. Cancer Therm..

[73] Zhang Y.R., Zhang M., Jiang Y.F., Li X.L., He Y.L., Zeng P.J., Guo Z.H., Chang Y.J., Luo H., Liu Y. (2018). Lentinan as an immunotherapeutic for treating lung cancer: A review of 12 years clinical studies in China. J. Cancer Res. Clin..

[74] Sakamoto J., Morita S., Oba K., Matsui T., Kobayashi M., Nakazato H., Ohashi Y. (2006). Meta-Analysis Group of the Japanese Society for Cancer of the Colon, R. Efficacy of adjuvant immunochemotherapy with polysaccharide K for patients with curatively resected colorectal cancer: A meta-analysis of centrally randomized controlled clinical trials. Cancer Immunol. Immunother..

[75] Taguchi T., Furue H., Kimura T., Kondo T., Hattori T., Itoh T., Osawa N. (1985). End point result of a randomized controlled study of the treatment of gastrointestinal cancer with a combination of lentinan and chemotherapeutic agents. Excerpta Med..

[76] Taguchi T., Furue H., Kimura T., Kondo T., Hattori T., Itoh T., Osawa N. (1985). End point results of phase III study of lentinan. Jpn. J. Cancer Chemother..

[77] Furue H., Kitoh I. (1981). Phase 111-study on Lentinan. Jpn. J. Cancer Chemother..

[78] Daba A.S., Ezeronye Q.U. (2003). Anti-cancer effect of polysaccharides isolated from higher basidiomycetes mushrooms Afr. J. Biotechnol..

[79] Furue H. (1985). Clinical-Evaluation of Schizophyllan(Spg) in Gastric-Cancer—Randomized Controlled-Studies. Int. J. Immunopharmacol..

[80] Fujimoto S., Furue H., Kimura T., Kondo T., Orita K., Taguchi T., Yoshida K., Ogawa N. (1984). Clinical-Evaluation of Schizophyllan Adjuvant Immunochemotherapy for Patients with Resectable Gastric-Cancer—A Randomized Controlled Trial. Jpn. J. Surg..

[81] Kimura Y., Mizuno H., Satake K., Tahara H., Tsukuda M. (1994). Clinical evaluation of Sizofiran an assistant immunotherapy in treatment of head and neck cancer. Acta Oto-Laryngol..

[82] Okamura K., Suzuki M., Chihara T., Fujiwara A., Fukuda T., Goto S., Ichinohe K., Jimi S., Kasamatsu T., Kawai N. (1986). Clinical-Evaluation of Schizophyllan Combined with Irradiation in Patients with Cervical-Cancer—A Randomized Controlled-Study. Cancer.

[83] Okamura K., Kinukawa T., Tsumura Y., Otani T., Itoh T., Kobayashi H., Matsuura O., Kobayashi M., Fukutsu T., Ohshima S. (1989). Adjuvant immunochemotherapy: Two randomized controlled studies of patients with cervical cancer. Biomed. Pharmacother..

[84] Miyazaki K., Mizutani H., Katabuchi H., Fukuma K., Fujisaki S., Okamura H. (1995). Activated (Hla-Dr+) T-Lymphocyte Subsets in Cervical-Carcinoma and Effects of Radiotherapy and Immunotherapy with Sizofiran on Cell-Mediated-Immunity and Survival. Gynecol. Oncol..

[85] Kamiyama Y. (1992). Improving effect of active hexose correlated compound (AHCC) on the prognosis of postoperative hepatocellular carcinoma patients. Eur. J. Surg. Res..

[86] Jones K. (1998). Maitake a patent medicinal food. Altern. Complement. Ther..

[87] Nanba H. (1997). Maitake D-fraction: Healing and preventive potential for cancer. J. Orthomol. Med..

[88] Nanba H., Kubo K. (1997). Effect of Maitake D-fraction on cancer prevention. Ann. N. Y. Acad. Sci..

[89] Deng G., Lin H., Seidman A., Fornier M., D’Andrea G., Wesa K., Yeung S., Cunningham-Rundles S., Vickers A.J., Cassileth B. (2009). A phase I/II trial of a polysaccharide extract from *Grifola frondosa* (*Maitake mushroom*) in breast cancer patients: Immunological effects. J. Cancer Res. Clin..

[90] Grinde B., Hedand G., Johnson E. (2006). Effects on gene expression and viral load of a medicinal extract from *Agaricus blazei* in patients with chronic hepatitis C infection. Int. Immunopharmacol..

[91] Twardowski P., Kanaya N., Frankel P., Synold T., Ruel C., Pal S.K., Junqueira M., Prajapati M., Moore T., Tryon P. (2015). A phase I trial of mushroom powder in patients with biochemically recurrent prostate cancer: Roles of cytokines and myeloid-derived suppressor cells for *Agaricus bisporus*-induced prostate-specific antigen responses. Cancer.

[92] Ahn W.S., Kim D.J., Chae G.T., Lee J.M., Bae S.M., Sin J.I., Kim Y.W., Namkoong S.E., Lee I.P. (2004). Natural killer cell activity and quality of life were improved by consumption of a mushroom extract, *Agaricus blazei* Murill Kyowa, in gynecological cancer patients undergoing chemotherapy. Int. J. Gynecol. Cancer.

[93] Tangen J.M., Tierens A., Caers J., Binsfeld M., Olstad O.K., Troseid A.M.S., Wang J.B., Tjonnfjord G.E., Hetland G. (2015). Immunomodulatory Effects of the *Agaricus blazei* Murrill-Based Mushroom Extract AndoSan in Patients with Multiple Myeloma Undergoing High Dose Chemotherapy and Autologous Stem Cell Transplantation: A Randomized, Double Blinded Clinical Study. Biomed. Res. Int..

[94] Valadares F., Garbi Novaes M.R., Canete R. (2013). Effect of Agaricus sylvaticus supplementation on nutritional status and adverse events of chemotherapy of breast cancer: A randomized, placebo-controlled, double-blind clinical trial. Indian J. Pharmacol..

[95] Fortes R.C., Recova V.L., Melo A.L., Novaes M.R.C.G. (2008). Effects of dietary supplementation with medicinal fungus in fasting glycemia levels of patients with colorectal cancer: A randomized, double-blind, placebo-controlled clinical study. Nutr. Hosp..

[96] Wesa K.M., Cunningham-Rundles S., Klimek V.M., Vertosick E., Coleton M.I., Yeung K.S., Lin H., Nimer S., Cassileth B.R. (2015). Maitake mushroom extract in myelodysplastic syndromes (MDS): A phase II study. Cancer Immunol. Immun..

[97] Griessmayr P.C., Gauthier M., Barber L.G., Cotter S.M. (2007). Mushroom-derived Maitake PET fraction as single agent for the treatment of lymphoma in dogs. J. Vet. Intern. Med..

[98] Yamaguchi Y., Miyahara E., Hihara J. (2011). Efficacy and Safety of Orally Administered *Lentinula edodes* Mycelia Extract for Patients Undergoing Cancer Chemotherapy: A Pilot Study. Am. J. Chin. Med..

[99] White R.W.D., Hackman R.M., Soares S.E., Beckett L.A., Sun B.X. (2002). Effects of a mushroom mycelium extract on the treatment of prostate cancer. Urology.

[100] Sumiyoshi Y., Hashine K., Kakehi Y., Yoshimura K., Satou T., Kuruma H., Namiki S., Shinohara N. (2010). Dietary Administration of Mushroom Mycelium Extracts in Patients with Early-Stage Prostate Cancers Managed Expectantly: A Phase II Study. Jpn. J. Clin. Oncol..

[101] Ito T., Urushima H., Sakaue M., Yukawa S., Honda H., Hirai K., Igura T., Hayashi N., Maeda K., Kitagawa T. (2014). Reduction of Adverse Effects by a Mushroom Product, Active Hexose Correlated Compound (AHCC) in Patients with Advanced Cancer During Chemotherapy-The Significance of the Levels of HHV-6 DNA in Saliva as a Surrogate Biomarker during Chemotherapy. Nutr. Cancer.

[102] Meera C.R., Janardhanan K.K. (2012). Antitumor Activity of a Polysaccharide-Protein Complex Isolated from a Wood-Rotting Polypore Macro Fungus *Phellinus rimosus* (Berk) Pilat. J. Environ. Pathol. Tox..

[103] Lee H., Cha H.J. (2018). *Poria cocos* Wolf extracts represses pigmentation in vitro and in vivo. Cell Mol. Biol..

[104] Hetland G., Tangen J.M., Mahmood F., Mirlashari M.R., Nissen-Meyer L.S.H., Nentwich I., Therkelsen S.P., Tjonnfjord G.E., Johnson E. (2020). Antitumor, Anti-Inflammatory and Antiallergic Effects of *Agaricus blazei* Mushroom Extract and the Related Medicinal Basidiomycetes Mushrooms, *Hericium erinaceus* and *Grifola frondosa*: A Review of Preclinical and Clinical Studies. Nutrients.

[105] Venturella G., Ferraro V., Cirlincione F., Gargano M.L. (2021). Medicinal Mushrooms: Bioactive Compounds, Use, and Clinical Trials. Int. J. Mol. Sci..

[106] Oka S., Tanaka S., Yoshida S., Hiyama T., Ueno Y., Ito M., Kitadai Y., Yoshihara M., Chayama K. (2010). A water-soluble extract from culture medium of Ganoderma lucidum mycelia sup-presses the development of colorectal adenomas. Hiroshima J. Med. Sci..

[107] Costa Fortes R., Lacorte Recova V., Lima Melo A., Carvalho Garbi Novaes M.R. (2010). Life quality of postsurgical patients with colorectal cancer after supplemented diet with Agaricus sylvaticus fungus. Nutr. Hosp..

[108] Okamura H., Anno N., Tsuda A., Inokuchi T., Uchimura N., Inanaga K. (2015). The effects of *Hericium erinaceum* (Amyloban^®^ 3399)on sleep quality and subjective well-being among female undergraduate students: A pilot study. Pers. Med. Universe.

[109] Schneider I., Kressel G., Meyer A., Krings U., Berger R.G., Hahn A. (2011). Lipid lowering effects of oyster mushroom (*Pleurotus ostreatus*) in humans. J. Funct. Foods.

[110] Choudhury M.B.K., Rahman T., Kakon A.J., Hoque N., Akhtaruzzaman M., Begum M.M., Choudhury M.S.K., Hossain M.S. (2013). Effects of *Pleurotus ostreatus* on blood pressure and glycemic status of hypertensive diabetic male volunteers. Bangladesh J. Med. Biochem..

[111] Choudhury M.B.K., Hossain M.S., Hossain M.M., Kakon A.J., Choudhury M.A.K., Ahmed N.U., Rahman T. (2013). *Pleurotus ostreatus* improves lipid profile of obese hypertensive non-diabetic males. Bangladesh J. Mushroom.

[112] Ohno S., Sumiyoshi Y., Hashine K., Shirato A., Kyo S., Inoue M. (2011). Phase I Clinical Study of the Dietary Supplement, *Agaricus blazei* Murill, in Cancer Patients in Remission. Evid. Based Complement. Altern..

[113] Yoshimura K., Kamoto T., Ogawa O., Matsui S., Tsuchiya N., Tada H., Murata K., Yoshimura K., Habuchi T., Fukushima M. (2010). Medical mushrooms used for biochemical failure after radical treatment for prostate cancer: An open-label study. Int. J. Urol..

[114] Okuno K., Aoyama T., Oba K., Yokoyama N., Matsuhashi N., Kunieda K., Nishimura Y., Akamatsu H., Kobatake T., Morita S. (2018). Randomized phase III trial comparing surgery alone to UFT + PSK for stage II rectal cancer (JFMC38 trial). Cancer Chemother. Pharmacol..

[115] Miyake Y., Nishimura J., Kato T., Ikeda M., Tsujie M., Hata T., Takemasa I., Mizushima T., Yamamoto H., Sekimoto M. (2018). Phase III trial comparing UFT + PSK to UFT + LV in stage IIB, III colorectal cancer (MCSGO-CCTG). Surg. Today.

